# Speckle Noise Reduction in Digital Holography by 3D Adaptive Filtering

**DOI:** 10.3390/s25175402

**Published:** 2025-09-01

**Authors:** Andrey A. Kerov, Alexander V. Kozlov, Pavel A. Cheremkhin, Anna V. Shifrina, Rostislav S. Starikov, Evgenii Y. Zlokazov, Elizaveta K. Petrova, Vsevolod A. Nebavskiy, Nikolay N. Evtikhiev

**Affiliations:** Laser Physics Department, Institute for Laser and Plasma Technologies, National Research Nuclear University MEPhI (Moscow Engineering Physics Institute), Kashirskoe Shosse 31, 115409 Moscow, Russiaavshifrina@gmail.com (A.V.S.); ezlokazov@gmail.com (E.Y.Z.);

**Keywords:** digital holography, denoising, speckle noise, sensor, 3D filtering, image enhancement, multi-look technique, optical-digital technique, adaptive filtering, noise reduction

## Abstract

Digital holography enables the reconstruction of both 2D and 3D object information from interference patterns captured by digital cameras. A major challenge in this field is speckle noise, which significantly degrades the quality of the reconstructed images. We propose a novel speckle noise reduction method based on 3D adaptive filtering. Our technique processes a stack of holograms, each with an uncorrelated speckle pattern, using an adapted 3D Frost filter. Unlike conventional filtering techniques, our approach exploits statistical adaptivity to enhance noise suppression while preserving fine image details in the reconstructed holograms. Both numerical simulations and optical experiments confirm that our 3D filtering technique significantly enhances reconstruction quality. Specifically, it reduces the normalized standard deviation by up to 40% and improves the structural similarity index by up to 60% compared to classical 2D, 3D median, BM3D, and BM4D filters. Optical experiments validate the method’s effectiveness in practical digital holography scenarios by local and global image quality estimation metrics. These results highlight adaptive 3D filtering as a promising approach for mitigating speckle noise while maintaining structural integrity in digital holography reconstructions.

## 1. Introduction

Holography is a technique of recording both the amplitude and phase information of an object [1], distinguishing it from classical imaging methods, which capture only amplitude distribution. Initially, holograms were recorded on photosensitive plates using weakly coherent light sources. The advent of coherent laser sources enabled the production of high-quality holograms, leading to the widespread adoption of holography [2]. The development of digital recording and image processing techniques further advanced this field, giving rise to digital holography, where digital cameras replace photosensitive plates for image capture, and computer-generated holography. This digital approach has found applications in diverse areas such as information security and encryption [3], optical data archiving [4], holographic displays [5], beam profiling [6] and shaping [7], microscopy [8], material characterization [9], visible light [10] and terahertz imaging [11], object detection [12], and many others.

Despite these advances, holographic image recording methods face certain challenges. Besides the inherent noise of digital cameras [13], speckle noise [14]—a granular interference pattern arising from the coherent nature of holographic imaging—remains a significant factor in degrading image quality. Techniques for speckle noise reduction broadly fall into two categories: digital [15,16,17,18,19,20,21,22,23,24,25,26,27,28,29] and optical-digital [30,31,32,33,34,35,36,37,38] methods. Digital methods focus on the postprocessing of a single recorded hologram to mitigate noise, but often at the cost of reduced image contrast and limited effectiveness in handling 3D scene depth. In contrast, optical-digital approaches improve noise suppression by modifying the optical setup to capture multiple holograms with varying speckle patterns. Such modifications include camera [30] or object shifting [31], the use of diffusers [32], angular detuning [33], and other optical adjustments, though these can pose practical limitations.

While recent deep learning approaches [39,40,41] show promise, their requirement for large annotated datasets and substantial computational resources limits their practicality in many scenarios.

In this article, we introduce a novel optical-digital method that utilizes advanced 3D adaptive filtering techniques. Unlike conventional methods such as median or averaging filters, our approach applies a modified adaptive Frost filter to a 3D array of reconstructed holographic images formed from multiple uncorrelated holograms. This hybrid approach combines the multi-look principle with sophisticated 3D adaptive filtering, yielding superior noise reduction compared to traditional and state-of-the-art methods.

The structure of the paper is as follows. Section 2 discusses the theoretical background of the methods employed. Section 3 details the proposed 3D adaptive filtering algorithm for holographic data. Section 4 presents numerical experiments and their outcomes, followed by Section 5, which describes the optical experiments. Finally, the Conclusions summarize the key results.

## 2. Theory

### 2.1. Holography

A hologram is a recorded interference pattern of two beams [9], which are called the object beam *E_o_*, the wavefront of which has interacted with the object, and the reference beam *E_r_*, the wavefront of which stays unchanged. The intensity distribution in that case is given by

(1) $$I\left(x,y\right)={\left|E_{o}+E_{r}\right|}^{2}={\left|E_{r}\right|}^{2}+{\left|E_{o}\right|}^{2}+E_{r}^{*}E_{o}+E_{r}E_{o}^{*}.$$

Then, the image of the object can be obtained by multiplying the intensity distribution I(x,y) by the complex conjugate of the reference beam $E_{r}^{*}\left(x,y\right)$:

(2) $$I\left(x,y\right)E_{r}^{*}\left(x,y\right)={\left|E_{o}\left(x,y\right)\right|}^{2}E_{r}^{*}\left(x,y\right)+{\left|E_{r}\left(x,y\right)\right|}^{2}E_{r}^{*}\left(x,y\right)+\;+E_{o}\left(x,y\right){E_{r}^{*}}^{2}\left(x,y\right)+{\left|E_{r}\left(x,y\right)\right|}^{2}E_{o}^{*}\left(x,y\right).$$

During image reconstruction from a hologram, three diffraction orders typically arise. The zero-order diffraction, corresponding to the first two terms in Equation (2), represents undiffracted radiation and manifests as uniform illumination. The other two orders correspond to the informative object image and the undesired twin image [42]. The presence of zero-order and twin images is a significant limitation in many recording methods, as the intensity of the original radiation contributes to unwanted diffraction orders alongside the informative object image [43]. To address this, spatial separation of diffraction orders is commonly employed, enabling isolation and reconstruction of the informative wavefront [43].

With advances in computer technologies, a new branch of holography—discrete holography—has emerged, encompassing both digital and computer holography. Computer holography [44,45] involves synthesizing holograms of modeled objects that may not physically exist, followed by their optical reconstruction. In contrast, digital holography captures holograms using digital imaging methods, with subsequent reconstruction performed either computationally or optically [46]. The development of digital hologram recording and reconstruction techniques has opened new opportunities to improve image quality through purely digital processing.

The main factors degrading image quality in digital holography include digital camera noise [13], zero-order and twin images [47], and speckle noise [14]. Among these, speckle noise is typically the most significant contributor to degradation. It arises from the variability of the medium through which the laser radiation propagates during hologram formation, producing a random interference pattern of diffusely scattered light beams that is recorded by the camera alongside the useful signal. Speckle noise [48] is commonly quantified using the root mean square deviation [49]:

(3) $$\sigma_{sp}=CI,$$

where *C* is the speckle coefficient, which is a constant and can be experimentally estimated; *I* is the average intensity of the reconstructed image.

### 2.2. Speckle Suppression Methods

Speckle noise reduction methods can be broadly divided into two categories: digital and optical-digital approaches. Digital methods aim to filter a single recorded hologram using computational algorithms that suppress noise while preserving image details. Basic techniques include median filtering [23] and averaging [24], while more advanced methods utilize local statistical characteristics of the image. These include the Wiener filter [25], Lee filter [26], and Frost filter [27]. Further improvements have been achieved using more sophisticated approaches, such as the iterative techniques [15], mean-median filter [16], BM3D [28] and its modifications [17], BM4D [18], total variation [19], wavelet-based filtering [20], non-local means filtering [21], interpolation-filtering techniques [22], compressed sensing [29], and neighborhood filtering [50]. These advanced algorithms leverage spatial redundancy and correlations in holographic data to enhance noise suppression while maintaining structural integrity.

Optical-digital methods, in contrast, modify the hologram recording setup to vary the speckle pattern before computational processing. This hybrid approach often outperforms purely digital methods, particularly when multiple holograms with different speckle realizations are available for processing. Optical-digital approaches typically involve two main steps:

- Recording multiple holograms with varying speckle distributions. This is achieved through modifications to the optical setup, such as using diffuser filters [32], angular detuning [33], polarization detuning [34], or wavelength detuning [35];
- Computational postprocessing of the acquired hologram set to suppress noise and improve image quality.

The simplest form of this method is to average a stack of reconstructed images [33,34,35]. Some techniques also apply additional filtering to the averaged image [32,36]. A more advanced strategy, proposed in [37], involves filtering the entire 3D array of reconstructed images. The algorithm operates as follows:

- A 3D stack is formed from multiple reconstructed images with different speckle realizations;
- A filtration window of size [N, N, M] is defined, where N (odd) denotes the spatial dimensions within each image, and M is the number of images in the stack;
- For each voxel in the array, the median value within the window is computed and assigned to the corresponding pixel in the output image;
- This process is repeated until the entire image is filtered;
- This 3D filtering approach has shown a notable improvement in hologram quality compared to classical methods, as it effectively leverages both spatial and cross-frame information to suppress speckle noise.

### 2.3. Frost Filter

The Frost filter is a well-established adaptive filter for speckle noise reduction [51]. The filter’s adaptivity is dictated by the following algorithm:

1. A filtration window *I* of the image of size [N×N] pixels is selected, where the window size must be odd to ensure the central position of the filtered pixel ξ(x,y);
2. For the window *I*, the distances from the central pixel ξ(x,y) to neighboring pixels are calculated and placed in the matrix *S*. This approach allows for greater consideration of pixels closer to ξ(x,y) than those farther away;
3. The average value $\bar{I}$ and variation $\sigma^{2}\left(I\right)$ are calculated for the pixels in the window;
4. The weighting parameter is then calculated, taking into account the statistical deviations of pixel values in the filtration window:

(4) $$B=D\cdot \left(\frac{\sigma^{2}\left(I\right)}{\bar{I}}\right),$$

where D is the damping coefficient value, responsible for the smoothness of filtration. Increasing D increases the sharpness of high-frequency areas of the image but reduces the quality of noise filtration.

1. The pixel weight matrix $W=e^{-B\cdot S}$ is calculated based on the obtained parameters;
2. The filtered pixel value is calculated: $Y=\frac{\sum (I\cdot W)}{\sum W}$.

This filter is adaptive, preserving the sharpness of image edges during filtration.

## 3. The Proposed Method

In the context of digital hologram enhancement, locally weighted filters—such as the Frost filter [27]—demonstrate superior performance compared to simpler algorithms like median filtering or averaging. Traditional 3D median or average filtering offers a straightforward approach to processing volumetric data by computing the median or mean value of pixels within a fixed window. While effective at suppressing outliers caused by speckle noise, these methods do not account for local statistical variations, often leading to detail loss in areas with high-intensity gradients.

We propose a 3D speckle reduction algorithm by extending the classic Frost filter into the volumetric domain. Our modified filter operates on a 3D volume, capturing spatial correlations within each image and temporal correlations across the stack of reconstructions. This is accomplished by incorporating adaptive behavior based on local statistical properties, allowing the filter to adjust dynamically to different image regions.

In the proposed 3D algorithm, the Frost filter is modified as follows. For each spatial location (x,y), a 3D processing window of size W×W×N is defined, where W is the side length of the square window, and N is the number of reconstructed holograms.

The local mean and variance are computed across the full 3D window:

(5) $$\mu_{x,y}=\frac{1}{\left|W_{x,y}\right|}\sum_{u,v,n\in W_{x,y}}A\left(u,v,n\right),$$

(6) $$\sigma_{x,y}^{2}=\frac{1}{\left|W_{x,y}\right|}\sum_{u,v,n\in W_{x,y}}{\left(A\left(u,v,n\right)-\mu_{x,y}\right)}^{2},$$

where $A\left(u,v,n\right)$ is the intensity at position $\left(u,v\right)$ at slice n. The damping coefficient is then calculated as

(7) $$C_{x,y}=\alpha \frac{\sigma_{x,y}^{2}}{\mu_{x,y}^{2}+\varepsilon },$$

with α being the damping factor and ε a small constant to prevent division by zero.

The distance term is retained only in the spatial domain:

(8) $$d\left(u,v\right)=\sqrt{{\left(u-x\right)}^{2}+{\left(v-y\right)}^{2}},$$

since inter-slice geometric distances are not physically meaningful for the reconstruction of the same scene under different speckle realizations. Treating all slices equally ensures that speckle diversity is fully exploited, while spatial fidelity is preserved.

Accordingly, the weight is assigned to each voxel:

(9) $$w\left(u,v,n\right)=\exp\left(-C_{x,y}d\left(u,v\right)\right),$$

which is normalized as

(10) $$\tilde{w}\left(u,v,n\right)=\frac{w\left(u,v,n\right)}{\sum_{u^{\prime },v^{\prime },n^{\prime }\in W\left(x,y\right)}w\left(u^{\prime },v^{\prime },n^{\prime }\right)},$$

Finally, the filtered pixel value is computed as the weighted average:

(11) $$B\left(x,y\right)=\sum_{u,v,n\in W_{x,y}}\tilde{w}\left(u,v,n\right)A\left(u,v,n\right).$$

Boundary regions are handled by zero-padding the input stack.

**Figure 1:**
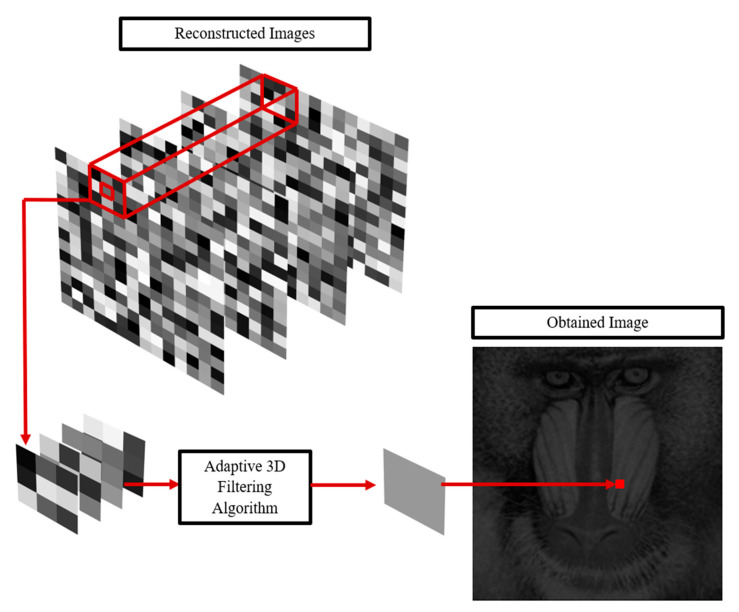
Schematic representation of the proposed 3D adaptive filtering method.

The algorithm consists of the following key steps:

- Recording a series of holograms with varying speckle distributions, enabled by modifying the optical setup;
- Reconstructing the holograms to form a 3D stack of images;
- Applying the modified adaptive Frost filter to the entire 3D dataset, leveraging cross-layer statistics for enhanced speckle reduction;
- A schematic overview of the proposed method is provided in Figure 1.

In summary, the proposed 3D Frost filtering approach enhances digital hologram reconstruction by incorporating both intra-layer and inter-layer statistical dependencies into a unified adaptive framework. Unlike conventional 2D methods, the 3D approach takes full advantage of volumetric multi-speckle data, enabling it to distinguish noise from true structural variations—even in regions with steep gradients—while preserving fine image details.

## 4. Numerical Experiments

**Figure 2:**
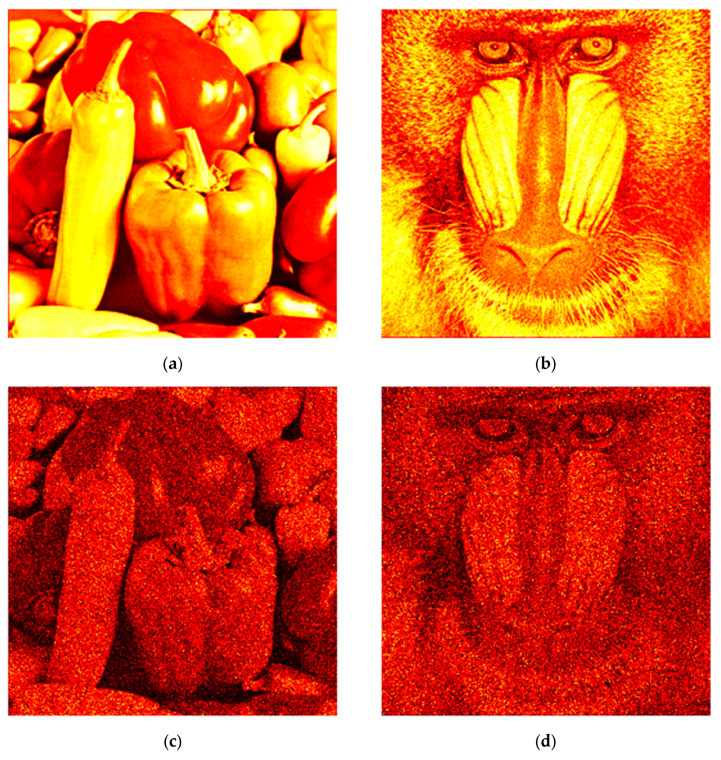
The test images (**a**,**b**) and reconstructed ones from the noisy holograms (**c**,**d**).

Initially, the proposed method, along with the standard and state-of-the-art algorithms, was evaluated through numerical experiments for speckle noise reduction. Test images “Peppers” and “Baboon”, each with a resolution of 256 × 256 pixels, are shown in Figure 2a,b, respectively. Holograms were synthesized using direct Fresnel diffraction calculations via the single-fast Fourier transform method [52]. To simulate a 3D stack, speckle noise was modeled according to the statistics of fully developed speckle, where the intensity $I_{s}$ follows a negative exponential distribution [48]:

(12) $$I_{s}=\frac{1}{\bar{I}}\exp\left(-\frac{I_{s}}{\bar{I}}\right),I_{s}\geq 0,$$

where $\bar{I}\;$ is the average value. Independent realizations of speckle were generated for each image, and the noise was applied in a multiplicative manner by scaling every pixel intensity of the hologram with the corresponding speckle factor.

For image reconstruction, inverse direct calculation of Fresnel diffraction was used. Examples of noisy images are shown in Figure 2c,d.

### 4.1. Reconstructed Image Quality Metrics

In addition to speckle contrast (see Equation (4)), the normalized standard deviation (NSTD) [53], the structural similarity index (SSIM) [54], blind/referenceless image spatial quality evaluator (BRISQUE) [55], and perception-based image quality evaluator (PIQE) [56] were used.

The NSTD is a pixel-by-pixel comparison method, while SSIM performs a comparison in a certain neighborhood of each pixel. Thus, a more accurate assessment of filtration quality can be obtained not only globally—since the statistical features of the image are spatially non-uniform—but also locally.

Each local SSIM assessment is calculated in a certain neighborhood of the investigated pixel [54]. The output values will range from −1 to +1. A value of +1 will be obtained in the case of the complete identity of the compared images. Mathematically, SSIM is expressed as follows:

(13) $$SSIM\left(x,y\right)=\frac{\left(2\mu_{x}\mu_{y}+C_{1}\right)\left(2\sigma_{xy}+C_{2}\right)}{\left(\mu_{x}^{2}+\mu_{y}^{2}+C_{1}\right)\left(\sigma_{x}^{2}+\sigma_{y}^{2}+C_{2}\right)}$$

where $\mu_{x}$ and $\mu_{y}\;$ are the average signal intensities, $\sigma_{x}$ and $\sigma_{y}$ are standard deviations, $\sigma_{xy}$ is covariance, $C_{1}$ and $C_{2}$ are constants.

NSTD is a common measure for assessing image quality and intensity distribution, in particular [53]

(14) $$NSTD={\left(1-\frac{{\left(\sum_{\xi ,\eta }^{M,N}E\left[\xi ,\eta \right]F\left[\xi ,\eta \right]\right)}^{2}}{\left(\sum_{\xi ,\eta }^{M,N}E^{2}\left[\xi ,\eta \right]\right)\left(\sum_{\xi ,\eta }^{M,N}F^{2}\left[\xi ,\eta \right]\right)}\right)}^{\frac{1}{2}}$$

where E and F are the original and reconstructed images, respectively; M, N are image dimensions; ξ,η  are the pixel numbers.

The NSTD value allows us to determine the degree of difference between images. The similarity assessment is based on the assumption that if the NSTD value is 0, the images are completely identical. If the NSTD value is close to 1, this indicates a significant difference between the images.

In addition to reference-based metrics (NSTD and SSIM), we also employed no-reference quality assessment methods, which allow evaluating images without the need for a ground truth. One such metric is the BRISQUE [55]. It is based on natural scene statistics in the spatial domain and quantifies deviations from the regular statistical properties typically observed in natural images. This makes the metric particularly useful for coherent imaging scenarios, where reference images are often unavailable, and subjective visual quality is of primary importance:

(15) $$BRISQUE\left(I\right)=f\left(v\left(I\right)\right),$$

where v(I) is the natural scene statistics-based feature vector of the image I, and f is a regression model trained on subjective quality scores. A lower value of BRISQUE indicates higher perceptual quality.

The PIQE [56] is another no-reference quality metric that extracts perceptually relevant features from different orientations and scales and classifies them into quality-aware categories. The final score is computed as a weighted aggregation of these feature-based distortions:

(16) $$PIQE\left(I\right)=\sum_{k}w_{k}d_{k}\left(I\right),$$

where $d_{k}\left(I\right)$ denotes the distortion feature in the category k, and $w_{k}$ is the corresponding perceptual weight. Similar to BRISQUE, lower PIQE values indicate better perceptual quality.

Together, BRISQUE and PIQE provide complementary perspectives on image fidelity, enabling robust evaluation of speckle suppression methods in both simulated and experimental holographic data.

### 4.2. Filtration Using the Proposed Method

To expand the analysis, various denoising methods were used:

- The classical Frost filter;
- The proposed 3D Frost method applied to multiple reconstructed images;
- The classical Lee filter;
- The newly developed 3D Lee method, applied to multiple reconstructed images;
- The 3D median method, applied to multiple reconstructed images;
- The BM3D filter;
- The BM4D filter, applied to multiple reconstructed images.

For the classical Frost filter [27], a 3 × 3 pixel window was used, as it provided optimal filtering performance while maintaining image sharpness. A damping factor of 3 was chosen to balance noise suppression and the preservation of high-frequency image details. The proposed 3D Frost filter used the same parameters to ensure fair comparability of the results. The Lee filter, a well-established adaptive filtering method based on local statistical analysis [26], was also considered. Both the classical Lee filter and the novel 3D Lee filter were implemented with a 3 × 3 window size. The 3D median filter, which has already demonstrated promising results in prior studies [37], was also included for comparison. For benchmarking purposes, the proposed method was further compared to state-of-the-art algorithms: conventional BM3D [28], which is applied to a single image, and BM4D [14], which is applied to a set of images.

**Figure 3:**
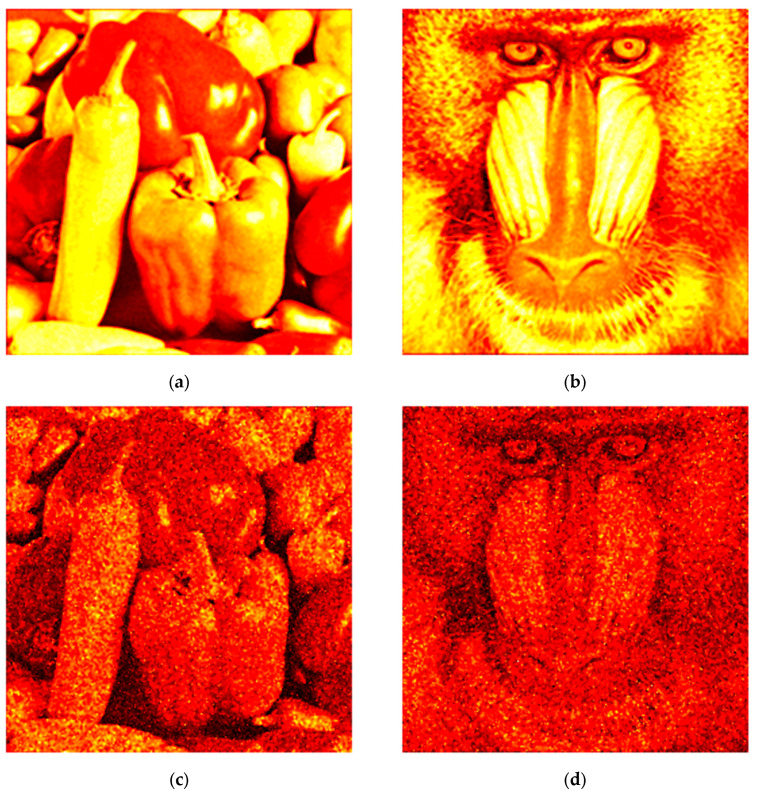

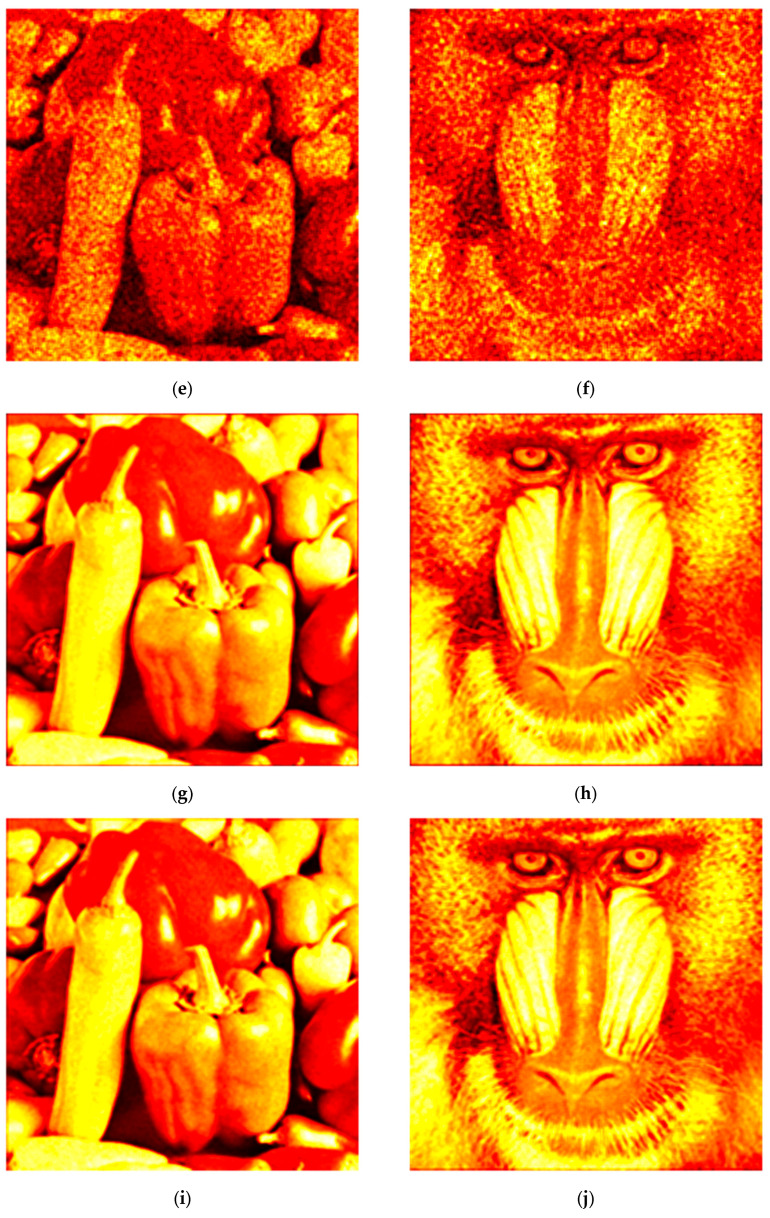

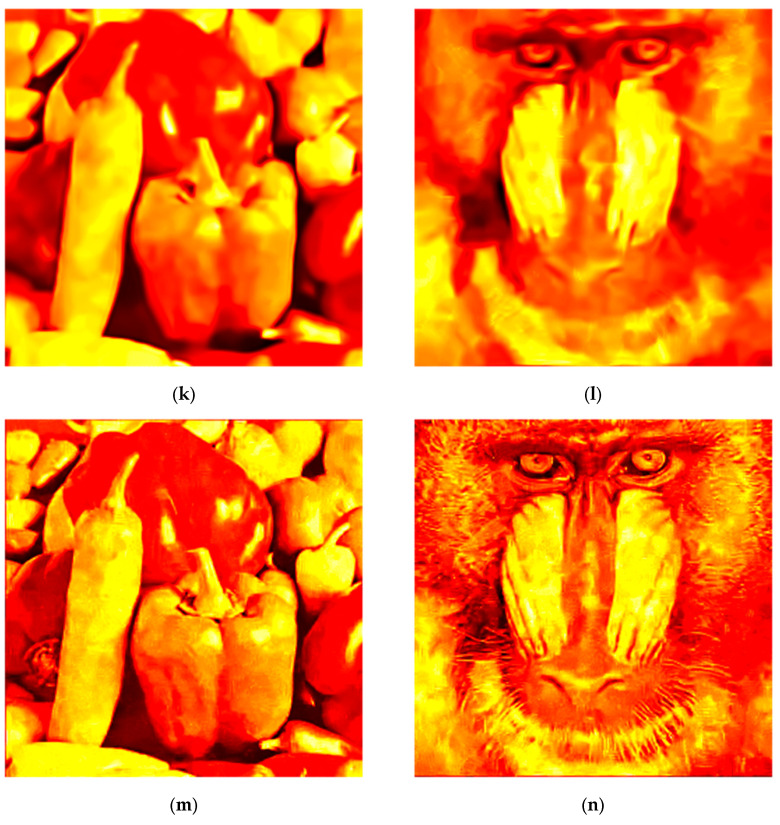
Results of filtering noisy images using the proposed (**a**,**b**), classical Frost (**c**,**d**), classical Lee (**e**,**f**), 3D Lee (**g**,**h**), 3D median (**i**,**j**), BM3D (**k**,**l**), and BM4D (**m**,**n**) methods.

**Figure 4:**
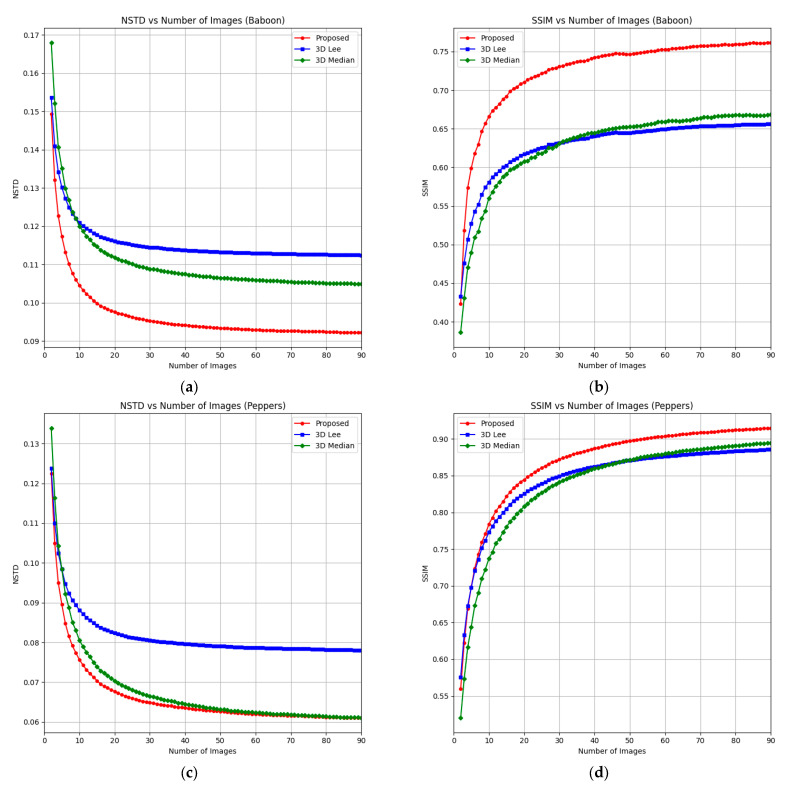
Dependencies of NSTD and SSIM vs. the number of reconstructed images (Baboon (**a**,**b**) and Peppers (**c**,**d**)) for the proposed, 3D Lee, and 3D median methods.

Filtered images obtained using each method are shown in Figure 3. For the 3D Frost, 3D Lee, 3D median, and BM4D filters, 90 reconstructed images were used. Quantitative metric values (NSTD and SSIM) for the filtered images are presented in Table 1, with the best results highlighted in bold. The dependencies of NSTD and SSIM on the number of images used in the 3D filtering window are illustrated in Figure 4. Additionally, images that were denoised by the proposed method using different numbers of holograms are given in the attached video files (Supplementary Materials).

Our method shows a significant improvement over the standard 2D Frost filter, reducing NSTD by over 60% and improving SSIM by over 160%. In contrast, reconstructed images using the standard Frost filter exhibit a high level of noise, highlighting the substantial advantage of the proposed 3D filtering approach over classical 2D methods.

Among the 3D filtering methods tested, the proposed 3D Frost method consistently delivered the best results. Compared to the 3D Lee and 3D median filters, it achieves roughly 20–30% improvements in NSTD and 10–35% improvements in SSIM, confirming that the 3D Frost filter is particularly effective for applications requiring strong noise suppression while preserving structural image details.

The dependency of SSIM and NSTD on the number of images in the 3D filtering window further illustrates the method’s efficiency. Remarkably, using only 10 images yields a 30% increase in SSIM and a 20% decrease in NSTD, reaching within 10% of the optimal values obtained using 90 images. Even with just five images, the proposed method often outperforms other filters applied to 90-image datasets. This efficiency demonstrates the algorithm’s ability to deliver near-optimal noise suppression and structural fidelity with minimal data and computational load. Unlike multi-look approaches that typically require hundreds of holograms, the proposed method achieves high-quality results with a significantly reduced number of inputs, making it particularly well-suited for time-sensitive applications.

In addition to outperforming classical and other 3D filters, the proposed method also surpasses state-of-the-art techniques such as BM3D and BM4D. As shown in Table 1, it achieves significantly lower NSTD and higher SSIM values across standard test images (e.g., Baboon and Peppers). The proposed method reduces NSTD by roughly 40% and 30% relative to BM3D and BM4D, respectively, while improving structural similarity by more than 50% and 65%.

To show the proposed method’s advantages over state-of-the-art techniques more clearly, Table 2 is added. The SSIM and NSTD values for several other test images are given for the proposed method and the BM4D filter. Processing times are also shown. Ninety holograms were used for both denoising methods. Numerical simulations were performed on a desktop computer equipped with an AMD Ryzen 5 5600X processor (6 cores and 12 threads), 16 GB DDR4 RAM operating at 4000 MHz, and an NVIDIA GeForce GTX 1660 Ti GPU with 6 GB of VRAM.

It can be seen that the proposed method consistently maintains superior noise suppression and structural preservation. Moreover, it delivers this performance with exceptional computational efficiency—being approximately 200 times faster than BM4D.

Thus, the proposed method outperforms various existing standard and state-of-the-art techniques. It provides significantly better quality of the reconstructed images. Additionally, compared to the multi-look approaches, high-quality results can be achieved with a significantly reduced number of holograms. Moreover, the method is significantly faster than the block matching with 3D and 4D filtering.

## 5. Optical Experiments

To demonstrate the practical applicability of the developed methods, a series of holograms with varying speckle distributions was recorded. An 80 mW, 561 nm laser (CNI Co., Changchun, China, model SSP-SLM-561-FN) was used as the radiation source. To ensure uniform beam distribution, a spatial low-pass filter—consisting of a micro-lens and a 50 μm aperture—was employed. Beam splitter 1 was used to divide the beam into reference and object beams.

To introduce variation in speckle distribution across the holograms, a rotating diffuser (∅24 mm, 2.3 mm thickness, 80–50 scratch-dig surface quality on the smooth side, and <3 arcmin parallelism) was placed in the object beam. After passing through the diffuser, the object beam was reflected off the sample. The beam splitter 2 was then used to recombine the object and reference beams. The resulting interference patterns were recorded using a Retiga R6 digital camera (QImaging, Surrey, BC, Canada) (5.9 MP, low noise [57]).

While our rotating diffuser arrangement induces temporal variation in the speckle field, it does not achieve full decorrelation between consecutive hologram frames. The Retiga R6 camera operates at only ~6.9 fps. This frame rate is far below the kilohertz-level rates typically needed to resolve fully independent speckle realizations; for instance, setups employing rotating diffusers have demonstrated effective speckle suppression at 2 kHz to 10 kHz [58]. Consequently, our imaging captures partial speckle averaging, which is beneficial but not sufficient for complete decorrelation. Nonetheless, even partial decorrelation contributes positively to speckle suppression. By tuning the diffuser’s rotational speed and the camera’s exposure time, it remains possible to integrate multiple quasi-independent speckle patterns per frame, resulting in reduced speckle contrast ($1/\sqrt{M}$ averaging law [59]).

To push speckle decorrelation further, alternative approaches such as ultra-high-speed cameras capable of kilohertz–megahertz frame rates or high-rate phase modulation methods (e.g., micro-electromechanical systems or digital micromirror devices [60]) can be used.

**Figure 5:**
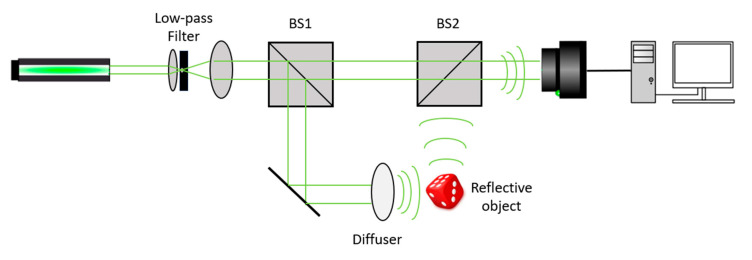
Experimental scheme for registering digital holograms with different speckle distributions.

**Figure 6:**
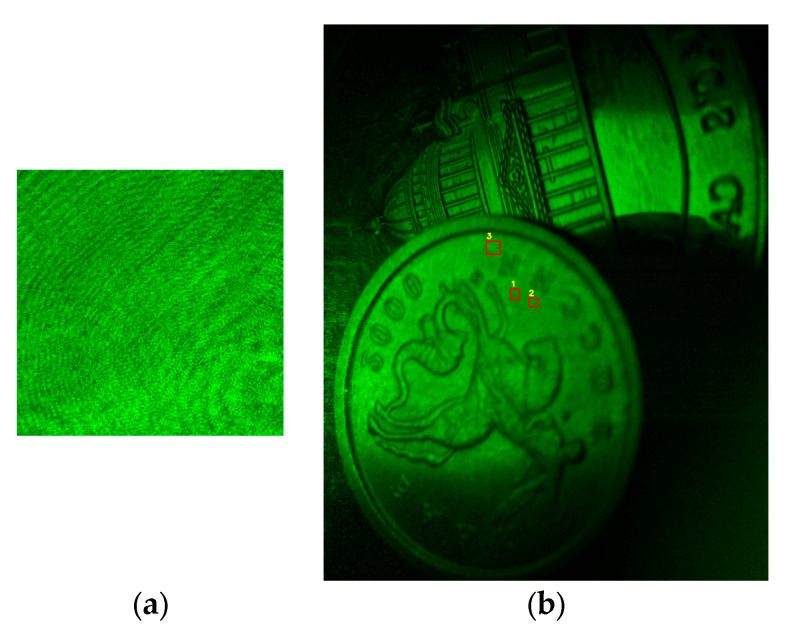
Example of a hologram (**a**) and a reconstructed image with several regions (1st, 2nd, and 3rd) for speckle contrast calculation (**b**).

A pair of closely placed coins was chosen as a reflective amplitude object for holograms recording. The experimental setup is illustrated in Figure 5, while an example of a digital hologram and the corresponding reconstructed image is shown in Figure 6. To evaluate speckle contrast values in the reconstructed images, uniform regions were selected for analysis. Several corresponding regions are shown with red rectangles in Figure 6b.

The speckle contrast was evaluated for images processed using BM4D, a widely adopted denoising method, and the proposed 3D Frost filter. The results for several zones and several numbers of images are presented in Table 3. For comparison, the values for unfiltered images were obtained by averaging over all frames within the designated regions of interest. Initial values for the speckle contrast are 0.52 ± 0.02 (zone 1), 0.53 ± 0.02 (zone 2), and 0.52 ± 0.01 (zone 3).

The performance of the two filters exhibits a clear and divergent trend as the number of input images increases. The BM4D filter demonstrates stable performance, with speckle contrast values showing minimal improvement beyond 20 images. In contrast, the proposed 3D Frost filter shows a strong, consistent improvement in speckle reduction. While values for the proposed method start with a higher speckle contrast at lower image counts (e.g., 10–20 images), its performance surpasses that of BM4D as more data are incorporated. For instance, with 60 images, the proposed method achieves a speckle contrast up to 58% lower than BM4D in certain zones. The average speckle contrast value for the proposed method was decreased by eight times to 0.064 ± 0.009, while for the BM3D filter, it was decreased to 0.108 ± 0.021.

This performance is further validated by the no-reference image quality metrics BRISQUE and PIQE, summarized in Table 4. The BRISQUE value for initial images is 40.3 ± 0.2. The initial PIQE value is 47.8 ± 0.7. Lower scores for these metrics indicate higher perceptual quality. The BM4D filter again shows consistent, stagnant scores across all image counts. For the proposed method, increasing the number of images ultimately leads to significantly better scores. This indicates that the proposed method not only reduces speckle noise more effectively but also does so while preserving significantly better image quality and structural integrity, especially with larger datasets.

**Figure 7:**
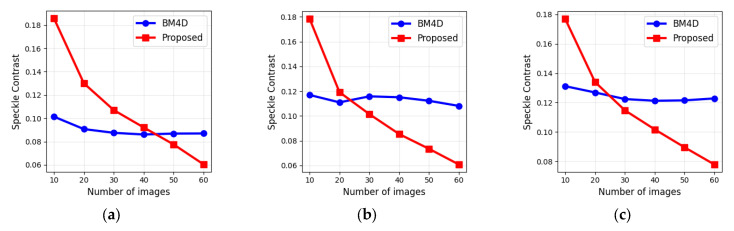
Dependences of speckle contrast in 1st (**a**), 2nd (**b**), and 3rd (**c**) zones vs. the number of images.

**Figure 8:**
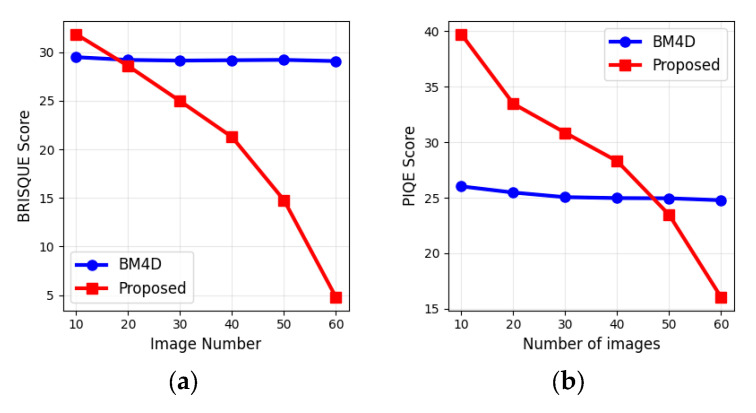
BRISQUE (**a**) and PIQE (**b**) scores dependences vs. the number of images.

Further insights into algorithm performance are provided in Figure 7, which illustrates examples of the dependences of speckle contrast on the number of input images, and in Figure 8, which shows the variation of BRISQUE and PIQE under the same conditions.

**Figure 9:**
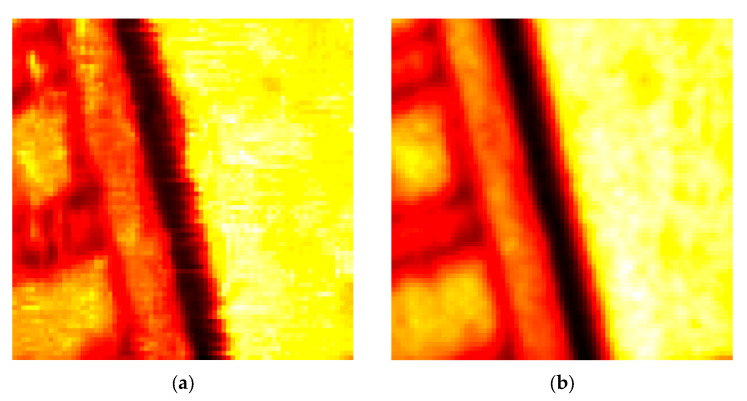
Enlarged areas of the images containing edges filtered by the BM4D (**a**) and the proposed filter (**b**).

A visual comparison of the filtered outputs confirms the structural preservation advantages of the proposed method, as initially indicated by its lower BRISQUE and PIQE scores. As shown in the enlarged areas of Figure 9, the BM4D filter tends to generate artifacts in regions with strong edges, degrading image clarity. The proposed filter avoids these artifacts, resulting in a more natural and structurally coherent image that is critical for accurate interpretation.

The robustness of BM4D and the proposed 3D Frost method was also compared using relative error. BM4D exhibited very low variability, with relative errors below 0.5% for global metrics and ranging from 0.6% to 15.8% for speckle contrast across regions of interest. The proposed method showed similar robustness in global metrics (BRISQUE: 0.2%; PIQE: 1.3%) and slightly higher variability for speckle contrast, with relative errors spanning approximately 1.4% to 16.1% depending on the zone. Overall, both methods demonstrate high stability.

In addition to quality improvements, the proposed filter significantly outperforms BM4D in terms of computational efficiency. Table 5 presents the processing times for both filters applied to 10, 20, 40, and 60 images. As the number of images increases, the performance gap becomes even more pronounced. The proposed method is up to 100 times faster than the BM4D filter. These processing times were calculated for the case when holograms were pre-restored before both methods were applied. This allows maximization of computational speed where memory is abundant. Under memory constraints, the time for the proposed method nearly doubles during the processing of dozens of images.

**Figure 10:**
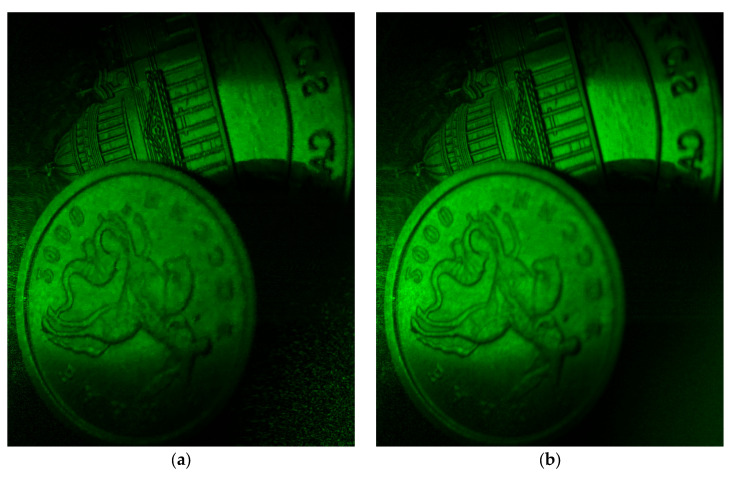
Images after filtration with BM4D (**a**) and the proposed (**b**) methods.

The images after filtration with BM4D and the proposed 3D Frost methods are shown in Figure 10. It can be seen that visually, speckle noise suppression was superior with the proposed filter, especially in the lower right corner of the image.

The optimal number of input holograms represents a trade-off between processing speed and reconstruction quality. A minimum of 10–15 holograms is sufficient for a perceptible improvement when speed is the priority. However, for applications demanding high quality, performance continues to improve with larger datasets, indicating that the highest quality is achieved by maximizing the number of holograms.

Thus, the proposed 3D Frost method demonstrates significantly better performance compared to existing techniques, including classical algorithmic variants. This makes the method highly effective for applications where acquiring multiple holograms is feasible. As more holograms are recorded, image quality continues to improve with additional holograms.

The primary limitation of the method lies in its requirement to register a sequence of holograms. Therefore, a balance must be found between the degree of image enhancement and the time required for hologram acquisition, as the scene should stay unchanged while recording. However, as shown in Figure 7 and Figure 8 and Table 3 and Table 4, only a small number of images are sufficient to achieve high-quality reconstruction.

Unlike traditional approaches, the proposed method incorporates a larger amount of signal information for calculating each pixel value, which contributes to a reduction in the required number of holograms. This, in turn, leads to an increase in the signal-to-noise ratio, further confirming the method’s suitability for practical applications.

Future improvements may include the integration of a dynamic weight function, adaptively adjusted based on inter-image variations or correlations within the 3D structure. This enhancement will be the subject of our further research in the field of digital hologram filtering.

## 6. Conclusions

In this work, we proposed and validated a 3D adaptive Frost filtering method for speckle noise reduction in digital holography. Numerical and optical experiments consistently confirm its advantages over both classical and state-of-the-art approaches. Compared to the conventional Frost filter, the proposed method reduces noise variation by more than a factor of two and more than doubles the structural similarity, highlighting the benefit of extending adaptive filtering into the volumetric domain. Relative to other 3D filters such as the median and Lee variants, our approach achieves roughly 20–30% stronger speckle suppression while providing up to one-third higher structural similarity, demonstrating a better balance between noise reduction and detail preservation.

Against BM3D and BM4D, the proposed filter yields about 30–40% lower noise measures and over 50% higher similarity indices while being nearly two orders of magnitude faster computationally. Optical experiments further show that as the number of holograms increases, the method continues to improve steadily: with 60 input frames, it achieves more than 50% lower speckle contrast compared to BM4D. No-reference perceptual metrics confirm this trend, with improvements of up to twofold in perceived image quality at higher frame counts.

A notable advantage of the proposed method is that it reaches near-optimal performance even with a modest number of holograms, as 10–15 images are already sufficient to achieve most of the possible quality gains, making it well-suited for practical, time-sensitive holographic imaging. These results demonstrate that 3D adaptive filtering not only surpasses traditional and advanced methods in speckle suppression and structural fidelity but also does so with exceptional efficiency. This makes the approach promising for applications in biomedical imaging, nondestructive testing, and optical metrology, where both image clarity and acquisition speed are critical.

## Figures and Tables

**Table 1:** Reconstructed image quality after filtering. The best results are highlighted in bold.

Filter	NSTD (Baboon)	SSIM (Baboon)	NSTD (Peppers)	SSIM (Peppers)
Frost	0.244	0.290	0.210	0.349
The Proposed Method	**0.092**	**0.762**	**0.061**	**0.915**
Lee	0.154	0.471	0.183	0.348
3D Lee	0.112	0.656	0.078	0.886
3D Median	0.122	0.580	0.105	0.669
BM3D	0.151	0.346	0.111	0.746
BM4D	0.118	0.392	0.109	0.618

**Table 2:** Metrics values for BM4D and the proposed method after filtration. The best results are highlighted in bold.

	BM4D			The Proposed Method		
Image	Processing Time, s	NSTD	SSIM	Processing Time, s	NSTD	SSIM
Cameraman	220	0.096	0.681	0.94	**0.067**	**0.867**
Lake	224	0.135	0.538	0.86	**0.079**	**0.890**
Walbridge	227	0.135	0.447	0.96	**0.095**	**0.833**

**Table 3:** Speckle contrast values for the reconstructed image. The best results are highlighted in bold.

Number of Images	Zone	BM4D	The Proposed Method
10	1	**0.101**	0.186
	2	**0.117**	0.178
	3	**0.131**	0.177
20	1	0.091	0.130
	2	0.111	0.119
	3	0.127	0.134
40	1	**0.086**	0.092
	2	0.115	**0.085**
	3	0.121	**0.102**
60	1	0.087	**0.060**
	2	0.108	**0.061**
	3	0.123	**0.078**

**Table 4:** BRISQUE and PIQE values for the reconstructed image. The best results are highlighted in bold.

Number of Images	BRISQUE		PIQE	
	BM4D	The Proposed Method	BM4D	The Proposed Method
10	**29.5**	31.9	**26.0**	39.7
20	29.2	**28.6**	**25.5**	33.5
40	29.2	**21.3**	**25.0**	28.3
60	29.1	**4.8**	24.8	**16.0**

**Table 5:** Processing times for BM4D and the proposed method.

Number of Images	Processing Time, s	
	BM4D	The Proposed Method
10	216	17.7
20	522	18.16
40	1172	18.23
60	1781	18.24

## Data Availability

Data are contained within the article or Supplementary Material.

## Supplementary Materials

The following supporting information can be downloaded at: https://www.mdpi.com/article/10.3390/s25175402/s1, See Videos S1 and S2 for supporting content.

## Abbreviations

The following abbreviations are used in this manuscript:

BM3D	block-matching and 3D filtering
BM4D	block matching with 4D filtering
BRISQUE	blind/referenceless image spatial quality evaluator
NSTD	normalized standard deviation
PIQE	perception-based image quality evaluator
SSIM	structural similarity index measure
